# Supplementary material for: 3D Multi-Branched SnO2 Semiconductor Nanostructures as Optical Waveguides
Source: Materials (Basel). 2019 Sep 26;12(19):3148. doi: 10.3390/ma12193148 (PMC6804052; doi:10.3390/ma12193148)
Supplement: Supplementary file 1 [file materials-12-03148-s001.pdf]

materials

Supporting Information of

3D Multi-Branched SnO_2 Semiconductor Nanostructures as Optical Waveguides

Francesco Rossella [1,‡,*], Vittorio Bellani [1], Matteo Tommasini [2], Ugo Gianazza [3], Elisabetta Comini [4] and Caterina Soldano [4,§,*]

1 Dipartimento di Fisica, Università di Pavia, Via Bassi 6, 27100 Pavia, Italy; vittorio.bellani@unipv.it
2 Dipartimento di Chimica, Materiali e Ingegneria Chimica "G. Natta", Politecnico di Milano, Piazza Leonardo da Vinci, 32, 20133 Milano, Italy; matteo.tommasini@polimi.it
3 Dipartimento di Matematica "F. Casorati", Università di Pavia, Via Ferrata 1, 27100 Pavia, Italy; gianazza@imati.cnr.it
4 Dipartimento di Ingegneria dell'Informazione, Università di Brescia, via Branze 38, 25131 Brescia, Italy; elisabetta.comini@unibs.it
‡ Present address: NEST, Scuola Normale Superiore and CNR-Istituto Nanoscienze, 56127 Pisa, Italy
§ Present address: Department of Electronics and Nanoengineering, Aalto University, Tietotie 3, 02150 Espoo, Finland
* Correspondence: francesco.rossella@sns.it (F.R.); caterina.soldano@aalto.fi (C.S.). These authors contributed equally.

1. Additional Raman Characterization

Figure S1 shows the room temperature Raman scattering spectrum of a 3D multi-branched semiconductor SnO_2 nanostructures, measured at λ = 632.8 nm with the laser spot focused on different locations along the entire nanostructure. Colors of Raman spectra are related accordingly to the location of the incident laser, as labelled in the images on the right. Raman signal corresponding to "node" location, whether primary or secondary ones, are the most amplified.

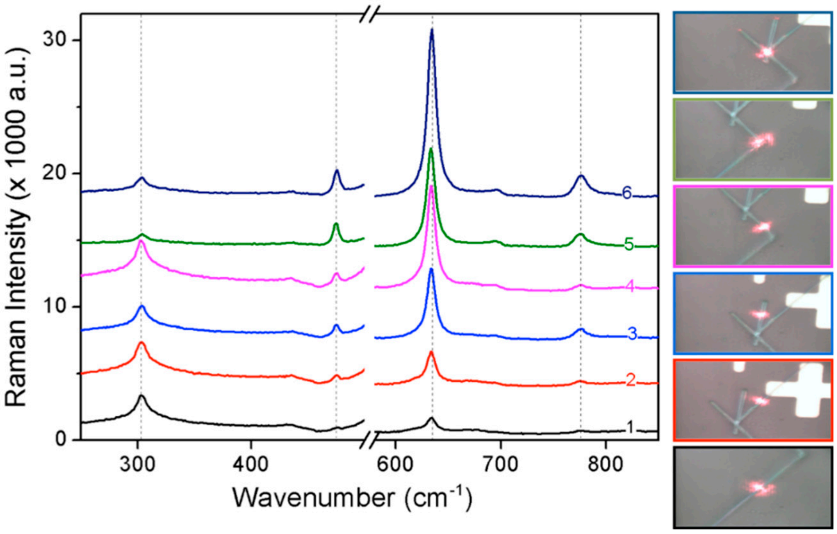

Figure S1. Room temperature Raman scattering spectrum of an individual and isolated straight SnO_2 nanowire, measured at λ = 632.8 nm with the laser spot focused on center wire regions (as labelled).

2. Integral Numerical Solution for Calculation of Interaction Volume

Let´s calculate the area E and the volume V of the region Ω, with Ω and E are defined as follows:

$$E = \{(x,y) \in \mathbb{R}^2 : x^2 + y^2 \leq r^2\}, \ \ \Omega = \begin{cases} x^2 + z^2 \leq R^2 \\ x^2 + y^2 \leq r^2 \end{cases}, r \leq R.$$

V is then given by:

$$V = 2 \iint_C \sqrt{R^2 - r^2} \ dx \, dy.$$

If we transform V in polar coordinates, we obtain:

$$\begin{aligned} V &= 2\int_{-\pi}^{\pi} d\theta \int_0^r \sqrt{R^2 - \rho^2 \cos^2\theta} \ \rho d\rho \ = \ 4\int_0^{\pi} d\theta \int_0^r \sqrt{R^2 - \rho^2 \cos^2\theta} \ \frac{\rho cos\theta d(\rho cos\theta)}{cos^2\theta} \\ &= \frac{4}{2}\int_0^{\pi} \frac{1}{cos^2\theta} \left[-\frac{2}{3}\left(R^2 - t^2\right)\right]_0^{rcos\theta} d\theta \ = \ \frac{4}{3}\int_0^{\pi} \frac{R^3 - \left(R^2 - r^2cos^2\theta\right)^{3/2}}{cos^2\theta} d\theta \\ &= \frac{4}{3}\int_0^{\pi} \frac{3R^4r^2 - 3R^2r^4cos^2\theta + r^6cos^4\theta}{R^3 + \left(R^2 - r^2cos^2\theta\right)^{3/2}} d\theta \\ &= \frac{4}{3}R^3 \int_0^{\pi} \frac{3a^2 - 3a^4cos^2\theta + a^6cos^4\theta}{1 + \left(1 - a^2cos^2\theta\right)^{3/2}} d\theta, \end{aligned}$$

where a is the ratio r/R, in the range $0 \leq a \leq 1$. Then $V = \frac{4}{3} R^3 \, I(a)$, with:

$$I(a) = \int_0^{\pi} \frac{3a^2 - 3a^4cos^2\theta + a^6cos^4\theta}{1 + \left(1 - a^2cos^2\theta\right)^{3/2}} d\theta.$$

I(a) can only be solved numerically, and through *quad* (*Matlab* routine) we obtain the following values:

Table S1. Integral numerical solution for Raman active volume.

a	*I(a)*
0	0
0.1	0.0471
0.2	0.1875
0.3	0.4193
0.4	0.7386
0.5	1.14
0.6	1.6162
0.7	2.1573
0.8	2.7498
0.9	3.3749
1	4
